# Self-medication among older adults in rural India: structural gaps in primary care and pain management

**DOI:** 10.1186/s12913-026-14114-z

**Published:** 2026-02-05

**Authors:** Suman Kanougiya, S. Bhaskarpandi, A. Surendhar, R. Dharani, R. Swetha, M. Dhanush, V. Oviya, B. Vishali

**Affiliations:** SRM School of Public Health (SPH), Faculty of Medicine and Health Sciences (MHS), SRM Institute of Science and Technology (SRMIST), Kattankulathur, Chengalpattu, Tamil Nadu 603203 India

**Keywords:** Self-medication, Older adults, Rural health, Primary care, Chronic disease, Injury, Health equity, India, Healthcare access, Health-seeking behavior

## Abstract

**Background:**

Older adults in rural India face persistent barriers to formal healthcare access, including financial constraints, transportation challenges, and limited geriatric-responsive services. In this context, self-medication often emerges as an informal strategy to manage symptoms and everyday health needs. Understanding the prevalence and correlates of self-medication among rural older adults is important for designing equitable, age-inclusive primary healthcare systems.

**Methods:**

A community-based cross-sectional survey was conducted between May and August 2024 among adults aged ≥ 55 years in five rural villages of Chengalpattu district, Tamil Nadu, India. Data were collected on sociodemographic characteristics, chronic morbidity, recent injuries, joint pain, prescribed medication use, and perceived barriers to care. Self-medication was assessed as any self-medication episode in the past three months (yes/no). Associations were examined using Poisson regression with robust standard errors to estimate adjusted prevalence ratios (aPRs).

**Results:**

Among 520 participants, 41.7% reported self-medication in the past three months. Although chronic morbidity was common, chronic illness burden was not independently associated with self-medication. In contrast, experiential health indicators—particularly recent injury (including fractures) and current joint pain—showed the strongest associations with self-medication in adjusted models. Adults aged ≥ 60 years and those currently married had a lower prevalence of self-medication compared with adults aged 55–59 years and those not currently married. Self-medication did not differ significantly by gender, caste, religion, or household income. Nearly one-third of participants reported irregular or no use of prescribed medications despite a high burden of chronic conditions.

**Conclusion:**

Self-medication is common among rural older adults in Tamil Nadu and appears more strongly associated with injury- and pain-related discomfort than with chronic disease diagnosis. This pattern is consistent with a symptom-driven pathway whereby acute discomfort, combined with barriers to timely and continuous care, is associated with unsupervised medication use. These findings highlight gaps in pain management, injury follow-up, and medication continuity within primary care and underscore the need for geriatric-responsive, equity-oriented outreach through Health and Wellness Centres/Ayushman Arogya Mandirs.

**Clinical trial registration:**

Not applicable.

**Supplementary Information:**

The online version contains supplementary material available at 10.1186/s12913-026-14114-z.

## Study highlights

**What is known:**

Self-medication is common in India, but evidence among rural older adults is limited.

Older adults face access barriers; unsafe self-medication can worsen inequities.

**What this study adds:**

41.7% prevalence of self-medication among rural adults aged ≥55 years.

Injury/fracture and joint pain were stronger correlates than chronic morbidity.

Minimal sociodemographic gradients; practices cut across caste/income/sex.

**Implications:**

Suggests a symptom-driven pathway under constrained access/continuity of care.

Prioritise pain management, injury follow-up, and medication continuity in PHC.

Use HWCs/Ayushman Arogya Mandirs for geriatric-responsive outreach and safety.

**Supplementary Information:**

The online version contains supplementary material available at 10.1186/s12913-026-14114-z.

## Introduction

### Population ageing and rural health system context in India

India is undergoing a rapid demographic transition. The population aged 60 years and older is projected to increase from about 100 million in 2011 to nearly 194 million by 2031, and to over 230 million by 2036, accounting for approximately 15–18% of the national population [1, 2]. This shift represents a fundamental restructuring of India’s population and places growing pressure on an already constrained health system. These pressures are particularly pronounced in rural areas, where access to continuous, comprehensive, and geriatric-responsive primary care remains limited [3–5]. Population ageing in India is occurring alongside a rising burden of non-communicable diseases (NCDs), multimorbidity, chronic pain, and functional decline [6]. Together, these trends intensify the demand for integrated, equitable, and person-centred models of care tailored to the needs of older adults [7, 8]. Nearly 70% of India’s older population resides in rural areas. Rural areas where structural disadvantages—including lower educational attainment, economic precarity, geographic isolation, weak transport infrastructure, and shortages of trained healthcare providers—compound barriers to timely and appropriate care [9–11].

### Burden of pain, injury, and chronic morbidity in later life

Nationally representative evidence highlights the substantial health burden faced by older adults in India. Data from the Longitudinal Ageing Study in India (LASI) indicate that 36.6% of adults aged 45 years and older are often troubled by pain, while 25.2% experience pain severe enough to limit usual activities. The prevalence of pain and pain-related disability is higher among women, rural residents, and socioeconomically disadvantaged groups [12]. Injury-related morbidity further contributes to health vulnerability in later life. Injuries account for approximately 10% of hospitalisations among adults aged 60 years and older, underscoring that injuries remain a significant but often under-recognised concern in ageing populations [13]. Despite this dual burden of chronic illness and injury-related discomfort, rural older adults frequently encounter fragmented primary care, limited access to pain management, and weak follow-up for chronic conditions [14, 15]. These gaps shape everyday health-seeking practices and treatment decisions, particularly for symptoms perceived as recurrent, manageable, or non-urgent.

### Defining self-medication and why it matters in older adults

Within this context, self-medication has emerged as a common coping strategy. The World Health Organization (WHO) defines self-medication as the selection and use of medicinal products by individuals to treat self-recognised symptoms or conditions without professional supervision [16]. While self-medication is sometimes framed as an expression of autonomy, it carries well-documented public health risks. These include adverse drug reactions, masking of serious illness, inappropriate antibiotic use, delayed diagnosis, and avoidable morbidity [17]. These risks are amplified in older adults. Age-related physiological changes, multimorbidity, and polypharmacy increase vulnerability to inappropriate dosing, drug–drug interactions, and adverse outcomes—particularly when pain or injury symptoms are managed without clinical assessment. Unsupervised medication use among older adults can worsen chronic disease trajectories, increase adverse events, and deepen existing health inequities.

### Prevalence of self-medication in India

Evidence from India suggests that self-medication is widespread, although prevalence varies considerably by population and setting. Systematic reviews and community-based studies report prevalence estimates ranging from approximately 35% to over 65% across diverse groups [18–20]. Substantial rural–urban variation has been documented, reflecting differences in access to healthcare services, regulatory enforcement, and local health-seeking norms. A community-based study from Maharashtra reported an overall self-medication prevalence of 29.1%, with markedly higher prevalence in urban areas (51.5%) compared with rural settings (7.7%) [17]. Conversely, other studies suggest that self-medication prevalence may be higher in rural areas where access to formal healthcare is constrained and medicines are readily available through informal channels [21]. These variations highlight the importance of context-specific analyses rather than uniform assumptions about self-medication behaviour.

### Self-medication among older adults: patterns and drivers

Although self-medication is often examined in younger or urban populations, multiple studies document substantial levels among older adults. Community-based studies in India indicate that self-medication prevalence among older adults ranges from 36% to 66%, particularly in settings with limited access to continuous and geriatric-responsive care [20, 22]. An urban multi-city study involving 600 older adults found that 19.7% reported self-medication, frequently alongside polypharmacy and potentially inappropriate prescribing. Self-medication was more common among individuals living alone, experiencing multimorbidity, or with recent hospitalisation, and commonly involved analgesics, paracetamol, and antibiotics [21]. Reliance on previous prescriptions, advice from pharmacists, financial constraints, and distance to healthcare facilities are consistently reported drivers of self-medication among older adults. These patterns suggest that self-medication in later life is shaped not only by individual preferences but also by structural and health-system constraints. Urban studies that triangulate self-reported medication use with prescription review and pill verification further demonstrate that unsafe medication practices among older adults reflect gaps in medication literacy and continuity of care rather than individual negligence [23]. Comparable evidence from rural settings remains limited, despite potentially greater structural constraints and fewer safeguards against inappropriate medicine use.

### Self-medication as a structural and health-system outcome

Importantly, self-medication among older adults should not be interpreted solely as an individual behavioural choice. Rather, it reflects broader systemic gaps in primary healthcare delivery. These include inadequate pain management, limited continuity of care for chronic conditions, diagnostic delays, and poor geriatric responsiveness within primary health systems [7, 10]. National initiatives such as Ayushman Bharat Health and Wellness Centres, Aayush Arogya Mandirs, and the National Programme for Health Care of the Elderly aim to strengthen comprehensive primary care. However, evidence suggests that older adults—particularly those in rural and socioeconomically vulnerable contexts—often remain peripheral to these reforms [24, 25]. While insurance coverage may increase hospital utilisation, it does not necessarily translate into preventive, continuous, or geriatric-responsive care for everyday health needs [13].

## Conceptual framework

**Fig. 1:**
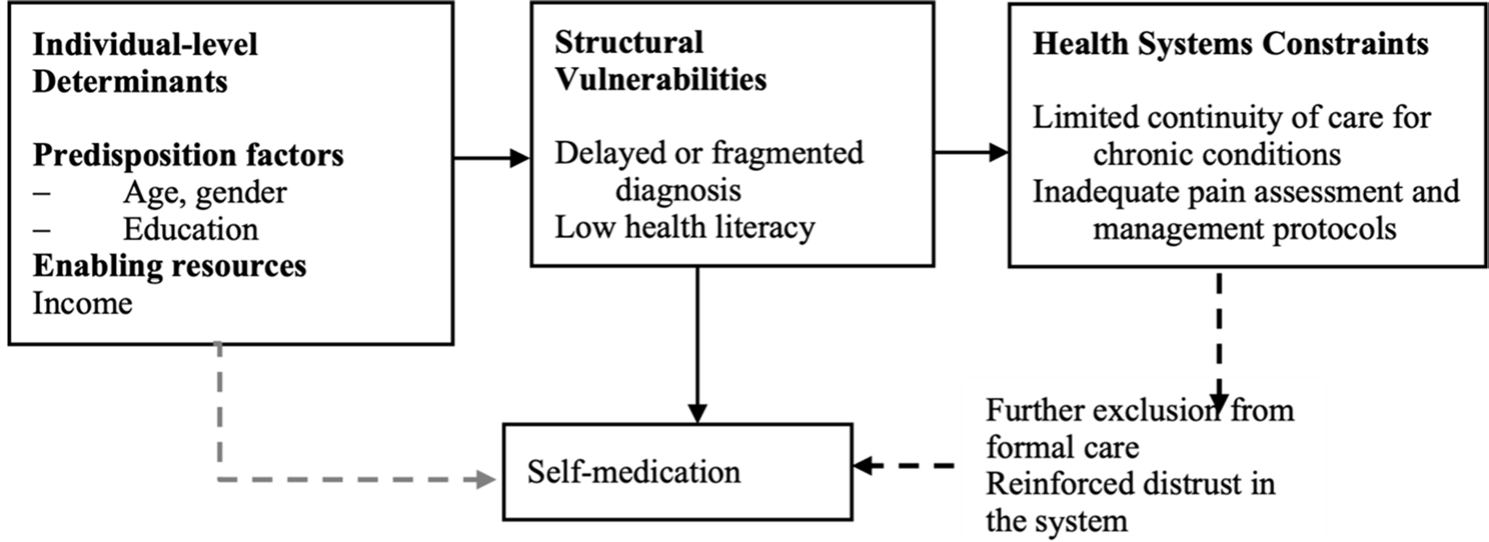
Integrated conceptual model linking health system gaps and structural vulnerabilities to self-medication practices among older adults. This conceptual model integrates Andersen’s behavioral model of health services use with a structural vulnerability and health-equity lens to illustrate how self-medication among older adults arises from the interaction of perceived health needs (particularly pain and injury), enabling constraints, and limitations in geriatric-responsive primary care. Self-medication is conceptualised as an adaptive and often supplementary response within constrained health system contexts, rather than as a simple disengagement from formal care

To conceptualise these dynamics, this study draws on three intersecting frameworks. Andersen’s Behavioral Model of Health Services Use [26] explains healthcare utilisation as shaped by predisposing characteristics (e.g., age, gender), enabling resources (e.g., income, transport, service availability), and perceived need (e.g., pain, injury, symptom urgency). The Structural Vulnerability Framework situates these behaviours within broader systems of inequality, highlighting how rurality, economic dependence, caregiving burdens, and the normalisation of pain constrain access to formal care. A Health Equity Lens further positions self-medication as a structural outcome of exclusion from timely, affordable, and geriatric-responsive primary care rather than as disengagement from the health system. Together, these frameworks underscore how self-medication among older adults may function as an adaptive—though potentially harmful—response within constrained health system contexts (Fig. 1).

## Study rationale and objectives

Despite a growing literature on self-medication in India, important gaps remain. Few studies have examined self-medication among older adults using an equity-informed framework that explicitly considers injury-related discomfort, pain, and structural barriers to care, particularly in rural settings. Adults aged 55–59 years—representing a transitional stage of ageing marked by emerging multimorbidity, pain, and shifts in help-seeking behaviour—are also under-represented or inconsistently analysed in national datasets. As a result, existing evidence provides limited insight into how structural vulnerabilities shape self-medication practices in later life, especially outside urban contexts. Accordingly, this study examines the prevalence of self-medication and its correlates among adults aged ≥ 55 years residing in five rural villages of Tamil Nadu. It assesses associations between self-medication and injury history, joint pain, chronic morbidity, sociodemographic characteristics, and perceived barriers to formal healthcare access. By interpreting these patterns through a structural vulnerability and health equity lens, the study aims to generate evidence to inform elder-responsive and equity-oriented primary healthcare strategies in rural India.

## Methods

### Study design, setting, and sample

This community-based cross-sectional study was conducted between May and August 2024 in five rural villages of Chengalpattu District, Tamil Nadu, India. Chengalpattu represents a rural–peri-urban interface with limited access to geriatric-responsive services, despite broader improvements in the state health system [4]. Tamil Nadu is undergoing rapid population ageing: 13.6% of the population were aged 60 years and above in 2021, with projections indicating an increase to 18.2% by 2031—the second-highest proportion among Indian states [1, 2]. Rural areas disproportionately host older adults with lower educational attainment and poorer socio-economic conditions compared with urban settings [27]. These populations also experience limited health-system infrastructure. Together, these disparities underscore the need for community-based research on ageing, health, and structural vulnerability.

Study sites were selected from 12 villages adopted under the Unnat Bharat Abhiyan (UBA) initiative [16, 28]. Villages were purposively shortlisted based on demographic characteristics and the willingness of local leaders to facilitate access, resulting in the final selection of five UBA villages for data collection.

Adults aged ≥ 55 years were included to capture a transitional stage of ageing that is often underrepresented in national datasets. Although the Longitudinal Ageing Study in India (LASI) operationally defines older age from 45 years onward, adults aged 55–59 years are inconsistently analysed in population-based research despite representing a critical life stage when multimorbidity, pain-related conditions, and shifts in help-seeking behaviour commonly begin to emerge [29–32]. Inclusion of this age group also reflects an evolving policy and research shift in India and other low- and middle-income countries toward recognising adults aged 55 years and older as part of the broader “older adult” population for public health planning and service delivery planning [33].

A multistage sampling approach was employed. Within each selected village, trained field investigators identified a random starting point and conducted consecutive household visits along residential lanes. All eligible adults within visited households were invited to participate. Eligibility criteria included: (a) age ≥ 55 years; (b) residence in the village for at least six months; and (c) ability to provide informed consent. Proxy interviews were not conducted. Older adults who were unable to provide informed consent due to apparent severe cognitive impairment or functional limitations were specified as an exclusion criterion. Formal cognitive or functional assessments were not conducted; therefore, exclusion was based on field-level judgement during recruitment. As a result, the potential underrepresentation of the most frail older adults is acknowledged as a study limitation.

This study formed part of a group Master of Public Health (MPH) dissertation on ageing and wellbeing. The sample size was estimated using Cochran’s formula, assuming a prevalence of 50% and a 5% margin of error, with a 20% adjustment for non-response [34]. During fieldwork, 625 adults aged ≥ 55 years were approached, of whom 521 completed the interview (response rate: 83.4%). For the present self-medication analysis, one participant with missing outcome data was excluded, resulting in a final analytic sample of 520. The most common reasons for non-participation were temporary absence or unavailability of eligible participants at the time of household visits.

### Data collection procedures

Because a complete and up-to-date sampling frame was not available prior to fieldwork, households were selected using a consecutive sampling approach starting from a randomly selected point along the main residential lane in each village. Field teams approached visibly occupied residential dwellings; shops, storage structures, and locked houses were excluded.

To minimize interviewer discretion and within-household clustering, only one eligible participant was interviewed per household. When more than one eligible adult (≥ 55 years) resided in a household, selection followed a pre-specified, non-discretionary algorithm: the eldest eligible member was approached first; if unavailable or unwilling to participate, the next eligible member was invited. Adults unable to provide informed consent were not interviewed, and proxy interviews were not conducted. Written informed consent was obtained from all participants; for individuals with limited literacy, the consent form was read aloud in Tamil and consent was documented using a thumb impression in the presence of a witness. Data collection was conducted between May and August 2024, aligned with the students’ approved semester break/on-duty period, and recruitment ceased once the target sample size was reached. Seasonal factors such as agricultural work patterns, heat, and intermittent weather-related mobility constraints may have influenced household availability and participation and are acknowledged as limitation in sampling.

Seven trained MPH student-investigators who also had proficiency in Tamil language and were familiar with cultural and context, conducted face-to-face structured interviews in Tamil. Training covered rapport building, neutral probing, research ethics, privacy safeguards, sensitive interviewing related to ageing, gender, stigma, and mental health, as well as electronic data capture procedures. Interviews were conducted in private settings (e.g., verandas or quiet rooms within the household); interviews were paused or relocated if privacy was interrupted. Participants who exhibited distress or reported unmet health needs were referred to local health workers or the nearest primary health centre. Data quality assurance procedures included daily field debriefings, routine supervisor review of completed forms, random call-backs, and spot field verifications. All responses were captured on password-protected electronic devices using KoboToolbox (https://www.kobotoolbox.org/*)* [35], with built-in range checks and skip logic to reduce entry errors. Data were encrypted and synchronized daily to a secure institutional server.

### Survey instrument, translation, and piloting

Data were collected using a structured questionnaire covering sociodemographic characteristics, health and wellbeing (self-reported chronic conditions, joint pain, and recent injuries), medication use (prescribed and non-prescribed), health-seeking behaviour, and access barriers. Recall periods were three months for medication use and twelve months for injury history. An English master questionnaire was developed specifically for this study and adapted for use in rural Tamil Nadu through a double forward–backward translation process [36]. Translations were reviewed by a bilingual expert panel and refined through cognitive interviews with older adults to ensure clarity, cultural relevance, and conceptual equivalence. A pilot test (*n* = 15) conducted in a non-study village led to minor wording simplifications and item re-ordering to improve flow and comprehensibility among low-literacy respondents. No items were removed during piloting. Cross-cultural adaptation followed recommended guidelines for translation and instrument refinement [36, 37]. The final questionnaire was administered in Tamil.

## Measures

### Primary outcome: self-medication

Self-medication was assessed using a two-step question sequence. First, participants were asked about their intended first response to health problems: *“If you are experiencing any health problems*,* what is the FIRST action that you will take?”* Response options included doing nothing; self-medication using home or ayurvedic remedies; self-medication using allopathic medicines; relying on advice from relatives, friends, or media; requesting medicines directly from a pharmacist; or seeking care from a government or private health facility. Second, participants were asked about *actual self-medication behaviour* using a frequency-based question: *“How many times have you self-medicated in the past three months?”* with response options never, once, twice, thrice, or more than three times [38].

For the primary analysis, self-medication was operationalised based on the frequency item to capture realised behaviour rather than intention. Participants reporting at least one episode of self-medication in the past three months were coded as having engaged in self-medication (yes = 1), while those reporting never were coded as no (0). The ordinal frequency categories were retained for descriptive analyses to characterise the recurrence and intensity of self-medication. Participants were instructed that self-medication included any self-initiated treatment undertaken without consulting a doctor, encompassing allopathic medicines, home remedies, Ayurveda, homeopathy, or medicines obtained directly from pharmacists, neighbours, or friends. Information on specific medication classes (e.g., analgesics, antibiotics, or herbal preparations) was not collected. This operationalisation aligns with the WHO definition of self-medication as the use of medicinal products or treatments by individuals to manage self-recognised symptoms or conditions without professional supervision [39] (p. 9), while preserving the temporal framing and behavioural specificity of the survey instrument. We did not collect a validated adherence scale or pill verification; future studies should incorporate adherence measures and medication review (e.g., pill packets or structured adherence tools).

### Health-related variables

Participants self-reported whether they had ever been diagnosed by a physician or health professional with the following conditions: hypertension, diabetes, asthma, kidney disease, heart disease, thyroid disorder, paralysis, and cancer (each coded Yes/No). A chronic illness index was constructed to capture multimorbidity and categorized as: none (0 conditions), single morbidity (1 condition), or co-/multimorbidity (≥ 2 conditions) [40]. This index was used to reflect overall chronic disease burden rather than condition-specific effects. Recent injury exposure was assessed by asking about injuries in the past 12 months and categorized as sprain/strain or fracture. In addition, current joint pain at the time of interview was recorded (Yes/No), given its relevance to pain-related self-management and informal care-seeking [41]. For selected conditions (e.g., hypertension, diabetes, asthma, kidney disease, heart disease, thyroid disorder, paralysis, cancer), respondents could also report whether they had not been tested in the past year, and these categories were retained descriptively to reflect gaps in routine screening.

### Prescribed medication use

Participants were asked whether they had taken any prescribed medications in the past three months. Those reporting use were further asked about regularity of intake, categorized as regular or irregular use; participants reporting no use were classified as not taking any medication. These responses were combined to create mutually exclusive categories of regular use, irregular use, or no prescribed medication use [42]. Formal medication adherence scales (including missed-dose frequency and reasons) were not administered.

### Sociodemographic and behavioural covariates

Sociodemographic variables included age (55–59, 60–74, ≥ 75 years), gender (male/female), marital or union status (currently married/in union vs. not), living arrangement (living alone vs. with others), education (no education; primary 1st–5th standard; middle and above ≥6th standard), caste/social group (SC/ST; OBC/MBC; none of the above), religion (Hindu; Muslim; Christian), work status in the past 12 months (yes/no), and monthly household income (≤₹5,000; ₹5,001–10,000; ≥₹20,000, as reported).

Behavioural variables included daily tobacco use (yes/no) and weekly alcohol consumption (yes/no).

## Statistical analysis

Descriptive statistics were used to summarise sociodemographic, health-related, and behavioural characteristics of the study population. Continuous variables are reported as means with standard deviations (SD), and categorical variables as frequencies and percentages. Bivariate associations with any self-medication in the past three months were examined using Pearson’s χ² tests for categorical variables and independent-samples *t*-tests for continuous variables [43]. Cross-tabulations were used to explore subgroup differences by age group, sex, marital status, chronic morbidity, recent injury, and joint pain.

Because the primary outcome—any self-medication in the past three months—was common (> 10%), adjusted prevalence ratios (aPRs) were estimated using Poisson regression with robust standard errors. This approach is recommended for cross-sectional studies with common outcomes, as odds ratios from logistic regression can overestimate relative associations and are non-collapsible [44, 45]. Although self-medication frequency was collected as an ordinal variable, regression analyses focused on a binary indicator (any vs. none) to reflect realised behaviour and to avoid misspecification arising from non-equidistant and right-censored response categories (e.g., “more than three times”). Ordinal regression models were explored but violated the proportional odds assumption; therefore, frequency data are presented descriptively only.

Covariate selection followed a combined conceptual and empirical strategy. Variables were pre-specified based on theoretical relevance, guided by Andersen’s Behavioral Model [26, 46] of Health Services Use and structural vulnerability frameworks. Bivariate analyses were used for descriptive purposes rather than sole variable selection. The final multivariable models adjusted for sociodemographic characteristics (age group, sex, marital/union status, living arrangement, religion, caste, education, household income), experiential health indicators (recent injury, joint pain, chronic morbidity burden), and behavioural factors (tobacco and alcohol use). Covariates were selected to control for confounding rather than to estimate mediation effects; therefore, structural access barriers were examined descriptively rather than included in regression models.

Multicollinearity was assessed by refitting the fully adjusted model as a linear probability model and computing variance inflation factors (VIFs), an accepted screening approach for generalized linear models. All VIFs were low (range: 1.05–1.76; mean: 1.36), indicating no evidence of problematic collinearity [47]. Model fit for the Poisson regression was evaluated using the Pearson goodness-of-fit statistic, which showed no evidence of overdispersion.

Missing data were minimal. Of the full sample (*N* = 520), 505 participants (97.1%) had complete data on the outcome and all covariates and were included in multivariable analyses. Missingness was primarily due to chronic morbidity status (*n* = 14) and injury type (*n* = 1). Comparisons between included and excluded participants showed no meaningful differences across key sociodemographic or health characteristics. Given the low and non-systematic level of missingness (< 3%), a complete-case analysis was undertaken [48]. Multiple imputation was not performed, consistent with methodological guidance indicating limited benefit under such conditions [49].

Several sensitivity analyses were conducted to assess robustness. First, a fully adjusted logistic regression model yielded directionally consistent estimates, reported as adjusted odds ratios (aORs) for comparison [50]. Second, alternative model specifications—including treating age as a continuous variable and fitting a parsimonious model restricted to age, injury, and joint pain—produced substantively similar results. Third, to address potential overlap between experiential health indicators, models were re-estimated after sequential exclusion of joint pain and injury variables; effect estimates for age and the remaining exposure remained stable. Fourth, discrimination of the logistic model was modest (ROC AUC ≈ 0.64) [51], which is typical for behavioural outcomes in population-based studies and supports an emphasis on effect size estimation rather than prediction [52]. All analyses were conducted using Stata version 15.1 [53]. Statistical tests were two-sided with a significance level of α = 0.05.

## Results

### Participant characteristics and bivariate analysis by self-medication status

As shown in the Table 1 among the 520 adults aged 55 years and above included in the analysis, 41.7% (*n* = 217) reported engaging in self-medication in the past three months, while 58.3% (*n* = 303) did not. The mean age of participants was 66.8 years (SD = 8.6). Age distribution did not differ significantly in bivariate analysis. Most respondents were women (70.4%), currently married (67.5%), and identified as Hindu (84.8%). Approximately one-third (34.4%) belonged to Scheduled Castes or Scheduled Tribes, and 61.0% to Other or Most Backward Classes (OBC/MBC). Nearly 40% had no formal education (40.4%), and 53.5% reported working in the past 12 months. Self-medication behaviour varied significantly by marital status (*p* = 0.047); a greater proportion of those not currently in a union reported self-medication (37.3%) compared to those currently married (62.7%). No significant associations were found for gender, caste, religion, education level, income category, or work status.

**Table 1:** Sociodemographic and health characteristics by self-medication status (analytic *N* = 520)

Characteristic	No self-medication *n* (%)	Yes self-medication *n* (%)	Total *n* (%)	Pearson χ² *p*-value†	Fisher’s exact *p*-value‡
Self-medication (past 3 months)	303 (58.3)	217 (41.7)	520 (100.0)	—	—
Age category (years)				0.118	0.119
55–59	53 (17.5)	54 (24.9)	107 (20.6)		
60–74	203 (67.0)	131 (60.4)	334 (64.2)		
≥ 75	47 (15.5)	32 (14.7)	79 (15.2)		
Gender				0.886	0.923
Male	89 (29.4)	65 (30.0)	154 (29.6)		
Female	214 (70.6)	152 (70.0)	366 (70.4)		
Marital status				0.047*	0.057
Not in union	88 (29.0)	81 (37.3)	169 (32.5)		
Currently in union	215 (71.0)	136 (62.7)	351 (67.5)		
Religion				0.882	0.921
Hindu	257 (84.8)	184 (84.8)	441 (84.8)		
Muslim	12 (4.0)	7 (3.2)	19 (3.7)		
Christian	34 (11.2)	26 (12.0)	60 (11.5)		
Caste / social group				0.331	0.330
SC/ST	110 (36.3)	69 (31.8)	179 (34.4)		
OBC/MBC	177 (58.4)	140 (64.5)	317 (61.0)		
Other	16 (5.3)	8 (3.7)	24 (4.6)		
Education				0.139	0.142
No education	128 (42.2)	82 (37.8)	210 (40.4)		
Primary (1–5th)	70 (23.1)	67 (30.9)	137 (26.3)		
Middle & above (≥ 6th)	105 (34.7)	68 (31.3)	173 (33.3)		
Worked in past 12 months				0.211	0.214
No	134 (44.2)	108 (49.8)	242 (46.5)		
Yes	169 (55.8)	109 (50.2)	278 (53.5)		
Monthly household income (INR)				0.230	0.233
≤ 5,000	108 (35.6)	87 (40.1)	195 (37.5)		
5,001–10,000	108 (35.6)	82 (37.8)	190 (36.5)		
≥ 20,000	87 (28.7)	48 (22.1)	135 (26.0)		
Tobacco use (Daily)				0.861	0.914
No	239 (78.9)	169 (78.2)	408 (78.6)		
Yes	64 (21.1)	47 (21.8)	111 (21.4)		
Weekly alcohol use				0.915	1.000
No	267 (88.1)	191 (88.4)	458 (88.2)		
Yes	36 (11.9)	25 (11.6)	61 (11.8)		

Percentages are column percentages within self-medication status (No vs. Yes). †Pearson’s χ² test; ‡Fisher’s exact test reported where expected cell counts were < 5. Item-level missingness: tobacco use and alcohol use *N* = 519 (1 missing). Statistical significance: *p < 0.05

Table 1. Distribution of sociodemographic and behavioural characteristics by self-medication status in the past 3 months among adults aged ≥ 55 years in rural Tamil Nadu (*N* = 520). Marital status showed a borderline association in bivariate testing (Pearson χ² *p* = 0.047; Fisher’s exact *p* = 0.057).

### Chronic conditions, injuries, and self-medication patterns

Chronic conditions were common in the study population but were not differentially distributed by self-medication status (Table 2). A small proportion reported not being tested in the past year. Overall, 71.9% of participants reported at least one chronic condition (364/506). The prevalence of any chronic condition was slightly higher among non-self-medicators (74.1%) than self-medicators (68.9%), although this difference was not statistically significant (χ² *p* = 0.326). Multimorbidity was frequent, with 32.8% reporting two or more chronic conditions, but was likewise not associated with self-medication status (χ² *p* = 0.326).

Hypertension (45.6%; 236/518) and diabetes (40.7%; 211/518) were the most commonly reported diagnoses. Neither condition showed a significant association with self-medication (hypertension: χ² *p* = 0.167; diabetes: χ² *p* = 0.612). Similarly, no significant differences by self-medication status were observed for asthma (*p* = 0.909), kidney disease (*p* = 0.842), heart disease (*p* = 0.206), thyroid disorder (*p* = 0.631), paralysis (*p* = 0.666), or cancer (*p* = 0.767); Fisher’s exact tests for sparse categories yielded consistent inferences.

In contrast, indicators of recent injury and pain were significantly associated with self-medication. Fractures in the past 12 months were more frequently reported among self-medicators (24.0%) than non-self-medicators (15.2%; valid *N* = 519), with statistically significant associations in both Pearson χ² and Fisher’s exact tests (χ² *p* = 0.012; Fisher *p* = 0.017). Current joint pain was also more prevalent among self-medicators (10.1%) compared with non-self-medicators (5.0%; *N* = 520), again supported by both χ² and Fisher’s exact tests (χ² *p* = 0.023; Fisher *p* = 0.025). Taken together, although chronic disease burden is widespread among older adults, acute injury and pain-related experiences—rather than the presence or number of chronic diagnoses—are more closely associated with self-medication behaviour.

**Table 2:** Bivariate associations between morbidity profile and self-medication in the past 3 months among older adults

Variable	Category	No self-medication *n* (%)	Yes self-medication *n* (%)	Total *n* (%)	Pearson χ² *p*	Fisher’s exact *p*
Chronic morbidity burden (*N* = 506)	No chronic condition	77 (25.9)	65 (31.1)	142 (28.1)	0.326	0.327
	Single morbidity	116 (39.1)	82 (39.2)	198 (39.1)		
	Co-/multi-morbidity	104 (35.0)	62 (29.7)	166 (32.8)		
Hypertension (*N* = 518)	No	148 (49.0)	122 (56.5)	270 (52.1)	0.167	0.156
	Yes	148 (49.0)	88 (40.7)	236 (45.6)		
	Never tested†	6 (2.0)	6 (2.8)	12 (2.3)		
Diabetes (*N* = 518)	No	165 (54.8)	128 (59.0)	293 (56.6)	0.612	0.653
	Yes	127 (42.2)	84 (38.7)	211 (40.7)		
	Never tested†	9 (3.0)	5 (2.3)	14 (2.7)		
Asthma (*N* = 518)	No	272 (90.1)	196 (90.7)	468 (90.4)	0.909	1.000
	Yes	26 (8.6)	18 (8.3)	44 (8.5)		
	Never tested†	4 (1.3)	2 (0.9)	6 (1.2)		
Kidney disease (*N* = 518)	No	286 (94.7)	202 (93.5)	488 (94.2)	0.842	0.835
	Yes	14 (4.6)	12 (5.6)	26 (5.0)		
	Never tested†	2 (0.7)	2 (0.9)	4 (0.8)		
Heart disease (*N* = 518)	No	274 (90.4)	195 (90.7)	469 (90.5)	0.206	0.213
	Yes	27 (8.9)	15 (7.0)	42 (8.1)		
	Never tested†	2 (0.7)	5 (2.3)	7 (1.4)		
Thyroid disorder (*N* = 519)	No	281 (92.7)	201 (93.1)	482 (92.9)	0.631	0.637
	Yes					

Values are n (%); percentages are column percentages within self-medication status (No/Yes)

p-values are from Pearson χ² tests; Fisher’s exact test is additionally reported where expected cell counts were < 5

†“Never tested in the past 1 year” categories are retained for completeness but should be interpreted cautiously due to small cell sizes

Valid N varies across variables due to item-level missingness

Table 2 Most diagnosed non-communicable diseases and overall multimorbidity were not associated with self-medication in bivariate analyses. In contrast, fracture in the past 12 months and current joint pain were significantly associated with a higher prevalence of self-medication in both Pearson χ² and Fisher’s exact tests, suggesting that acute injury and pain-related conditions may be more proximate drivers of unsupervised medicine use among older adults than chronic diagnoses alone.

### Self-medication across age groups by injury type in the past 12 months

**Fig. 2:**
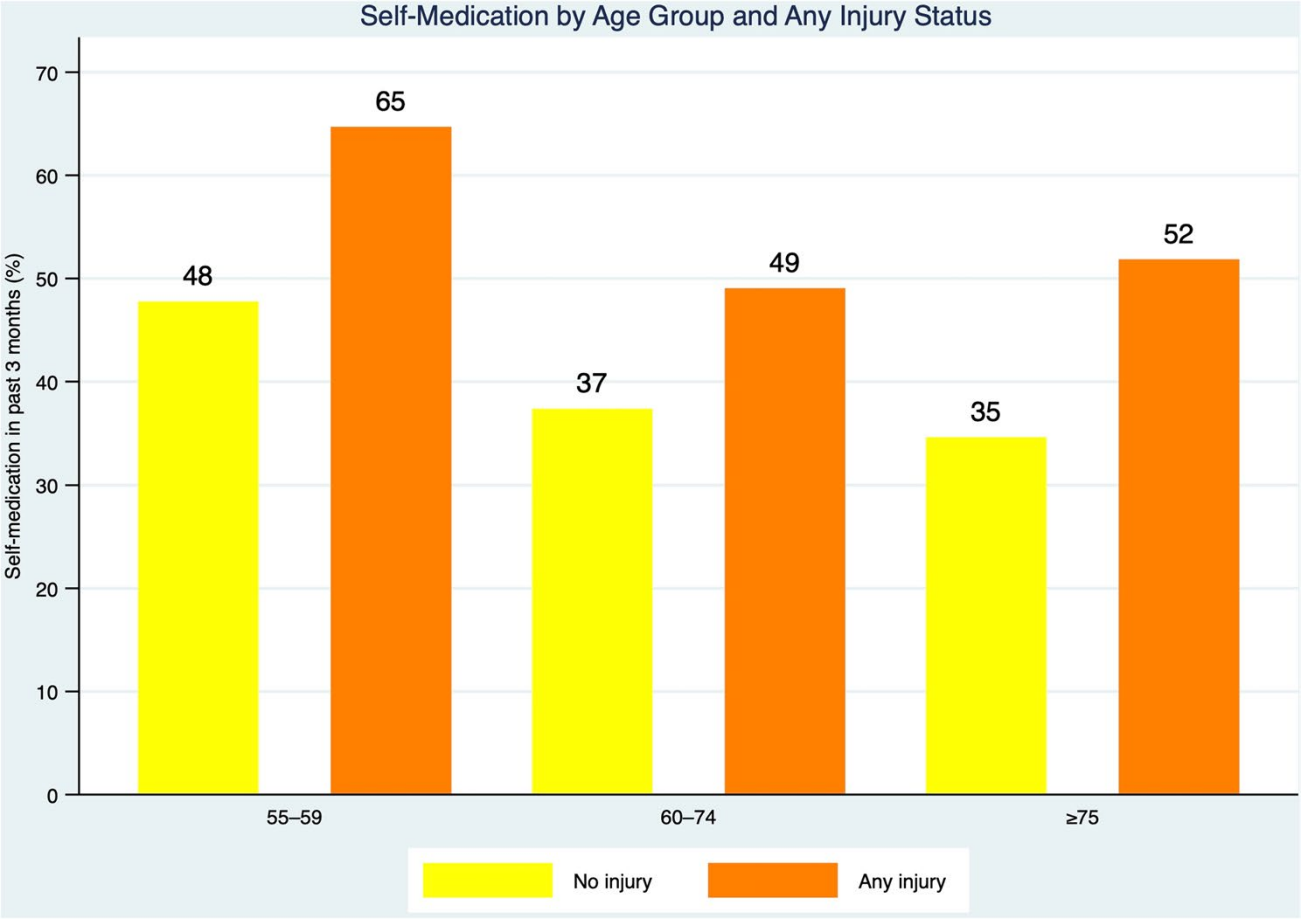
Self-medication prevalence by age group and any injury in the past 12 months. Bar chart showing the percentage of adults aged ≥ 55 years who reported any self-medication in the past three months, stratified by age group (55–59, 60–74, ≥ 75 years) and injury status in the past 12 months (any injury vs. no injury). Self-medication prevalence was higher among participants reporting any injury across all age groups, with the largest absolute difference observed among those aged 55–59 years. Bars represent mean prevalence (%) within each stratum; values are shown on bars

Figure 2 illustrates age-specific patterns in self-medication prevalence stratified by injury status in the past 12 months. Across all age groups, the proportion of older adults reporting self-medication in the past three months was consistently higher among those with a recent injury compared with those without injury. The difference was most pronounced in the youngest age group (55–59 years), where self-medication prevalence was substantially higher among individuals reporting any injury. Although overall self-medication prevalence declined with increasing age, the injury-related gradient persisted across age categories, indicating that recent injury remains an important correlate of self-medication even among the oldest adults. These descriptive patterns complement the bivariate analyses, reinforcing the interpretation that acute injury experiences—rather than age alone—are a key driver of self-medication behaviour among older adults.

### Prescription medication use and self-medication behaviour

Among respondents reporting a corresponding diagnosis, prescription medication use in the past three months was generally high but varied by condition (Table 3, Panel A). Slightly over half of participants with hypertension reported taking medication (53.4%; 119/223), while 59.4% (117/197) of those with diabetes reported current use. Treatment coverage was substantially higher for several other diagnosed conditions, including kidney disease (79.2%; 19/24), heart disease (85.4%; 35/41), wheezing or respiratory conditions (81.3%; 26/32), thyroid disorders (89.3%; 25/28), and paralysis (91.7%; 11/12). In contrast, among respondents reporting other illnesses specified through free-text responses, fewer than two in five reported taking prescription medication (38.9%; 14/36), suggesting that some of these conditions may be episodic or managed without sustained pharmacological treatment.

At the population level (*N* = 520), prescription medication use was common (Table 3, Panel B). Overall, 58.1% of participants reported regular prescription medication use, 12.3% reported irregular use, and 28.9% reported not taking any prescription medication; medication frequency data were missing for 0.8% of participants. Mutually exclusive medication-use categories were derived by resolving inconsistent responses through prioritisation of regular over irregular use and any use over non-use.

Self-medication in the past three months was reported by 41.7% of older adults, while 58.3% reported no self-medication (Table 3, Panel C). Among those who self-medicated, repeated use was common: 14.8% reported self-medicating twice, 8.1% thrice, and 6.0% four or more times during the reference period, indicating that self-medication was frequently recurrent rather than occasional.

Overall prescription medication use did not differ significantly between participants who did and did not self-medicate (68.1% vs. 69.2%; *p* = 0.848), nor did the proportion reporting no medication use at all (29.9% vs. 29.8%; *p* = 1.000). However, medication regularity differed by self-medication status. Irregular prescription medication use was significantly more common among participants who self-medicated compared with those who did not (17.1% vs. 10.7%; Fisher’s exact *p* = 0.049), while regular medication use did not differ significantly between groups (*p* = 0.279). Self-medication among older adults is associated primarily with intermittent or inconsistent treatment patterns rather than complete disengagement from prescribed care.

Condition-specific patterns of prescription medication use were broadly similar across self-medication groups for most diagnosed conditions, including hypertension, kidney disease, heart disease, paralysis, wheezing, thyroid disorders, and other reported illnesses (Table 4, Panel D). In contrast, among participants with diabetes, those who reported self-medication were less likely to be taking prescribed diabetes medication than their non-self-medicating counterparts (57.1% vs. 67.8%), consistent with a significant association observed in bivariate analyses (Fisher’s exact *p* = 0.045). This pattern suggests a selective gap in diabetes-specific management rather than a generalised avoidance of prescribed treatment.

**Table 3:** Prescription medication use and self-medication patterns among older adults in the past 3 months (*N* = 520)

**Panel A. Prescription medication use among respondents reporting the corresponding diagnosis**			
**Diagnosed condition**	**Not taking medication n (%)**	**Taking prescription medication n (%)**	**Total (diagnosed)**
Hypertension	104 (46.6)	119 (53.4)	223
Diabetes	80 (40.6)	117 (59.4)	197
Kidney disease	5 (20.8)	19 (79.2)	24
Heart disease	6 (14.6)	35 (85.4)	41
Paralysis	1 (8.3)	11 (91.7)	12
Wheezing / respiratory condition	6 (18.8)	26 (81.3)	32
Thyroid disorder	3 (10.7)	25 (89.3)	28
Other illness†	22 (61.1)	14 (38.9)	36
Total (condition–person observations)	227 (38.3)	366 (61.7)	593
**Panel B. Overall prescription medication regimen (mutually exclusive categories)**			
**Prescription medication regimen**	**n**	**%**	
Not taking any medication	150	28.85	
Irregular use	64	12.31	
Regular use	302	58.08	
Missing	4	0.77	
Total	520	100.00	
**Panel C. Self-medication frequency in the past 3 months**			
**Self-medication frequency**	**n**	**%**	
Never	303	58.27	
Once	67	12.88	
Twice	77	14.81	
Thrice	42	8.08	
Four or more times	31	5.96	
Total	520	100.00	
Derived binary indicator			
Any self-medication	n	%	
No	303	58.27	
Yes	217	41.73	
Total	520	100.00	
**Panel D. Self-medication prevalence among respondents reporting each diagnosis**			
**Diagnosed condition**	**No self-medication n (%)**	**Yes self-medication n (%)**	**Total (diagnosed)**
Hypertension	148 (62.71)	88 (37.29)	236
Diabetes	127 (60.19)	84 (39.81)	211
Kidney disease	14 (53.85)	12 (46.15)	26
Heart disease	27 (64.29)	15 (35.71)	42
Paralysis	8 (57.14)	6 (42.86)	14
Wheezing / respiratory condition	26 (59.09)	18 (40.91)	44
Thyroid disorder	20 (62.50)	12 (37.50)	32
Other illness†	23 (46.00)	27 (54.00)	50
Total (condition–person observations)	393 (60.00)	262 (40.00)	655

Values are n (%). Percentages in Panels A and D are row percentages calculated within each diagnosed condition

Denominators in Panels A and D are condition-specific; totals represent condition–person observations and do not correspond to unique individuals, as respondents may report multiple diagnoses

†Other illness derived from free-text responses

Self-medication refers to the use of medicines without a prescription in the past three months, including allopathic, traditional, or pharmacist-initiated medicines

In Panel B, mutually exclusive prescription medication categories were derived from three items (regular / irregular / none). Inconsistent responses were resolved by prioritising regular over irregular use, and any use over no medication

Values are n (row %); denominators are condition-specific

### First response to illness symptoms by chronic illness status

When asked about their first response to health problems, nearly one-third of participants (31.9%) reported a self-medication pathway—comprising home or ayurvedic remedies, allopathic self-medication, or requesting medicines directly from a pharmacist—while 62.3% reported seeking doctor-prescribed care. Visiting a private clinic or hospital was the most common first action (40.6%), followed by care-seeking at government facilities (21.7%). Smaller proportions reported doing nothing (5.6%) or relying on informal advice from relatives or friends (0.2%) (Table 4). In contrast, realised self-medication behaviour over the preceding three months was more prevalent: 41.7% of participants reported at least one episode of self-medication. This discrepancy suggests that informal medicine use often extends beyond initial care-seeking intentions and may reflect episodic or supplementary self-management alongside formal healthcare use.

When examined by chronic-illness status (valid *N* = 506), first-action patterns differed significantly (Table 4, Panel A). Participants without a chronic condition were more likely to delay or self-manage symptoms, including doing nothing (9.9%) or using ayurvedic or home remedies (22.5%), compared with those with a chronic condition (4.1% and 7.7%, respectively). In contrast, participants with chronic illness were more likely to seek facility-based care, particularly from private clinics or hospitals (44.2% vs. 31.7%) and government facilities (23.9% vs. 16.2%). Overall differences in first response by chronic-illness status were statistically significant (χ²(5) = 33.67, *p* < 0.001).

To further assess informal care-seeking, self-medication pathways (ayurvedic/home remedies, allopathic self-medication, and requesting medicines from pharmacists without a prescription) were collapsed into a single indicator (Table 4, Panel B). Using this definition, self-medication as the first response was significantly more common among participants without chronic illness (42.3%) than among those with chronic illness (27.8%), while reliance on non-self-medication pathways was higher among those with chronic conditions (72.3% vs. 57.8%) (χ²(1) = 9.91, *p* = 0.002).

**Table 4:** First response to illness symptoms by chronic illness status among older adults

**Panel A. First response/action to illness symptoms (overall distribution; ** ***N*** ** = 520)**			
**First response/action**	**n**	**%**	
Do nothing	29	5.58	
Self-medication – ayurvedic/home remedy	61	11.73	
Self-medication – allopathy	59	11.35	
Rely on advice from relatives/friends	1	0.19	
Request pharmacist without prescription	46	8.85	
Visit government hospital	113	21.73	
Visit private hospital/clinic	211	40.58	
Total	520	100.00	
**Panel B. First response/action by chronic illness status (valid ** ***N*** ** = 506)**			
**First response/action**	**No chronic illness (** ***n*** ** = 142) n (%)**	**Any chronic illness (** ***n*** ** = 364) n (%)**	**Total (valid ** ***N*** ** = 506) n (%)**
Do nothing	14 (9.86)	15 (4.12)	29 (5.73)
Self-medication – ayurvedic/home remedy	32 (22.54)	28 (7.69)	60 (11.86)
Self-medication – allopathy	12 (8.45)	43 (11.81)	55 (10.87)
Request pharmacist without prescription	16 (11.27)	30 (8.24)	46 (9.09)
Visit government hospital	23 (16.20)	87 (23.90)	110 (21.74)
Visit private hospital/clinic	45 (31.69)	161 (44.23)	206 (40.71)
Total	142 (100.00)	364 (100.00)	506 (100.00)
Pearson χ² (df = 5) = 33.67, *p* < 0.001			
**Panel C. Any self-medication as first action by chronic illness status (valid ** ***N*** ** = 506)**			
**First action was self-medication (any)**	**No chronic illness** **(** ***n*** **= 142)** **n (%)**	**Any chronic illness** **(** ***n*** **= 364)** **n (%)**	**Total** **(valid** ***N*** **= 506)** **n (%)**
No	82 (57.75)	263 (72.25)	345 (68.18)
Yes	60 (42.25)	101 (27.75)	161 (31.82)
Total	142 (100.00)	364 (100.00)	506 (100.00)

Pearson χ² (df = 1) = 9.91, *p* = 0.002

Percentages in Panels B and C are column percentages within chronic illness status

Chronic illness status was missing for 14 participants; therefore, Panels B and C are based on valid *N* = 506

Self-medication includes use of medicines without a prescription, encompassing allopathic drugs, traditional/ayurvedic remedies, and pharmacist-initiated medicines

Informal advice from relatives/friends was infrequently reported and is shown only in the overall distribution (Panel A)

Self-medication defined as ayurvedic/home remedy, allopathy, or pharmacist without prescription; values are n (column %)

Values are n (column %); column percentages are within chronic-illness status

Table 4 shows that private healthcare facilities were the most common first response to illness symptoms overall. However, respondents without chronic conditions were significantly more likely to initiate self-medication as their first action compared with those reporting any chronic illness, while individuals with chronic conditions were more likely to seek formal care—particularly private facilities—suggesting differential health-seeking pathways based on illness experience and continuity of care.

### Structural barriers to healthcare access

Structural barriers were examined descriptively to contextualise the findings rather than included as covariates, as they were conceptualised as potential mediators—rather than confounders—of the relationship between health status and self-medication behaviour. Respondents could report more than one access-related barrier. Financial (42%), distance-related (33%), and transport-related (34%) constraints were commonly reported, highlighting the structural context within which care-seeking decisions are made among rural older adults. Fewer than one-third of participants (29%) reported no access-related concerns, while a substantial proportion reported additional barriers such as lack of accompaniment (17%) or other challenges (6%) (Table S1).

### Model diagnostics and assessment of collinearity

#### Missing data and complete-case checks

Missingness in the analytic variables was minimal. Of the full sample (*N* = 520), chronic illness status was missing for 14 participants and injury type for one participant; all other covariates were complete. As a result, 505 participants (97.1%) had complete data for the outcome and all covariates and were included in multivariable analyses. Comparisons between included and excluded participants showed no substantive differences across key demographic or health characteristics (data not shown), supporting the use of a complete-case approach. Given < 3% missingness and no evidence of differential missingness on key predictors, we did not perform MI; MI is unlikely to materially change estimates under these conditions (Supplementary Table S2).

#### Multicollinearity

Potential multicollinearity among predictors was assessed using variance inflation factors (VIFs) derived from an ordinary least squares regression including all covariates from the main Poisson model. VIF values were uniformly low (range: 1.05–1.76; mean VIF = 1.36), indicating no evidence of problematic multicollinearity.

Goodness-of-fit for the Poisson regression with robust standard errors was assessed using the Pearson chi-square statistic, which indicated adequate model fit (Pearson χ² = 295.9, *p* = 1.00), with no evidence of overdispersion. Model stability was further assessed using an events-per-variable (EPV) criterion. The main model included 34 non-intercept parameters and 209 outcome events, yielding an EPV of approximately 6.1, which is acceptable for exploratory multivariable analyses with robust variance estimation. Findings were consistent across sensitivity analyses using logistic regression models and alternative specifications (Supplementary Table S2).

#### Assessment of collinearity between joint pain and injury

Given the conceptual overlap between joint pain and recent injury, we conducted a targeted assessment of collinearity between these two experiential health variables. Cross-tabulation with chi-square and Fisher’s exact tests showed a statistically significant but weak association between joint pain and injury type (sprain/strain vs. fracture) (χ²(1) = 4.78, *p* = 0.029; Fisher’s exact *p* = 0.046). The effect size was small (Cramér’s V = 0.096), indicating minimal shared variance (Supplementary Table S3). Together with low VIF values for both variables, joint pain and injury capture related but distinct dimensions of health experience and can be retained simultaneously in multivariable models without concern for meaningful multicollinearity.

#### Multivariable analysis of factors associated with self-medication

Bivariable (unadjusted) associations between individual covariates and any self-medication in the past three months are presented in Supplementary Table S4 and are reported for descriptive purposes only; these analyses were not used for variable selection. At the bivariate level, self-medication was significantly more common among participants reporting joint pain (OR = 2.17, 95% CI: 1.10–4.28) and among those who had sustained a fracture in the past 12 months compared with those reporting a sprain or strain (OR = 1.75, 95% CI: 1.13–2.73). Participants aged 60–74 years were significantly less likely to report self-medication than those aged 55–59 years (OR = 0.63, 95% CI: 0.41–0.98), and being currently in a marital or union relationship was associated with lower odds of self-medication (OR = 0.69, 95% CI: 0.47–1.00). Other socio-demographic characteristics—including chronic illness status, gender, living arrangement, religion, caste, employment status, income, tobacco use, and alcohol use—were not significantly associated with self-medication in bivariable analyses. Educational attainment showed a marginal association, with higher odds of self-medication among those with primary education compared with no schooling.

**Fig. 3:**
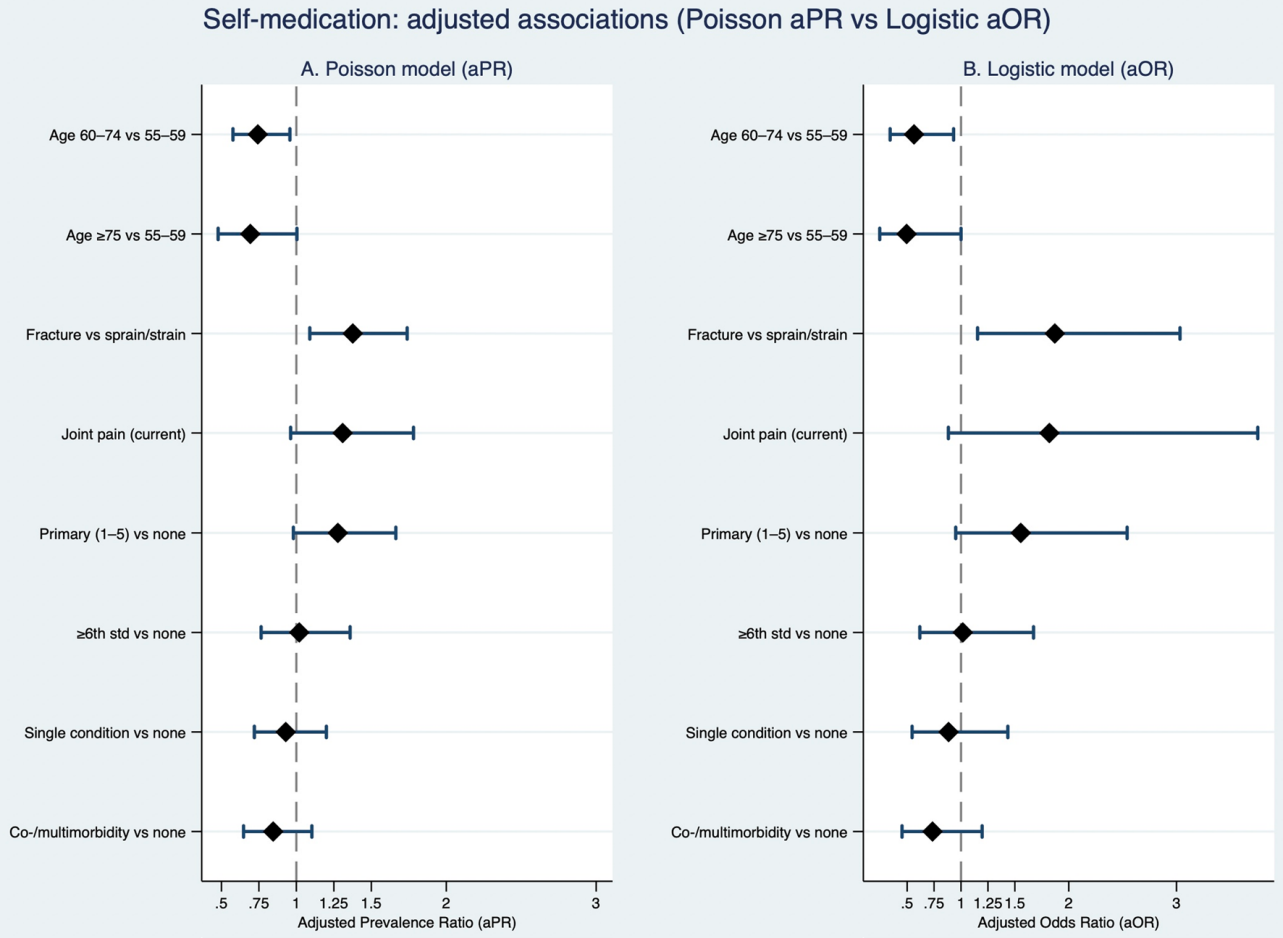
Adjusted associations with self-medication in the past three months. Panel A displays adjusted prevalence ratios (aPRs) estimated using Poisson regression with robust standard errors, and Panel B displays adjusted odds ratios (aORs) from a sensitivity logistic regression model. Both models examine associations between health status, pain and injury indicators, and socio-demographic characteristics with any self-medication in the past three months. Points represent adjusted effect estimates and horizontal lines denote 95% confidence intervals; the vertical dashed line indicates the null value (aPR or aOR = 1.0). Models were adjusted for chronic illness status (none, single, co-/multimorbidity), joint pain, injury type (fracture vs. sprain/strain), sex, age group (55–59 [reference], 60–74, ≥ 75), marital or union status, living arrangement, religion, caste, education, work in the past 12 months, household income, tobacco use, and alcohol use (complete-case *N* = 505)

Multivariable analyses were conducted using both logistic regression (adjusted odds ratios, aOR) and Poisson regression with robust standard errors (adjusted prevalence ratios, aPR) on a complete-case analytic sample (*N* = 505) (Table S4). After adjustment for chronic illness status, pain and injury indicators, and socio-demographic characteristics, recent injury and age remained independently associated with self-medication. Participants reporting a fracture in the past 12 months had significantly higher likelihood of reporting self-medication compared with those reporting a sprain or strain (aOR = 1.87, 95% CI: 1.14–3.07; aPR = 1.38, 95% CI: 1.09–1.74). Age showed a consistent inverse association: compared with adults aged 55–59 years, those aged 60–74 years (aOR = 0.56, 95% CI: 0.35–0.92; aPR = 0.74, 95% CI: 0.58–0.96) and those aged ≥ 75 years (aOR = 0.50, 95% CI: 0.25–0.99; aPR = 0.69, 95% CI: 0.48–1.00) were significantly less likely to report self-medication (Fig. 3).

Joint pain remained positively associated with self-medication in adjusted models, but this association did not reach conventional levels of statistical significance (aOR = 1.82, 95% CI: 0.89–3.73; aPR = 1.31, 95% CI: 0.96–1.78). Chronic illness status and other socio-demographic variables—including gender, marital or union status, living arrangement, religion, caste, education, employment status, income, tobacco use, and alcohol use—were not independently associated with self-medication after adjustment. Overall, experiential health factors—particularly recent injury severity and age—are more salient correlates of self-medication than chronic disease status or socio-demographic characteristics once covariates are considered simultaneously.

#### Sensitivity analyses

Sensitivity analyses were conducted to assess the robustness of the main findings to alternative model specifications, potential overlap between experiential health variables, and analytic assumptions. First, a fully adjusted logistic regression model with robust standard errors was estimated using the same covariate set as the primary Poisson model. The direction and magnitude of associations were consistent with the main analysis, indicating that inferences were robust to model specification (odds ratios vs. prevalence ratios). In particular, fracture in the past 12 months remained associated with higher self-medication, while older age groups showed consistently lower likelihood of self-medication compared with adults aged 55–59 years. Joint pain showed a positive but attenuated association after adjustment. These results were concordant with the primary multivariable findings presented in Fig. 3.

Second, to examine whether associations were driven by overlap between experiential health indicators, the Poisson model was re-estimated after sequentially excluding joint pain and, separately, injury status. When joint pain was excluded, fracture in the past 12 months remained significantly associated with higher self-medication prevalence (aPR = 1.41, 95% CI: 1.12–1.78). Conversely, when injury status was excluded, joint pain remained independently associated with higher self-medication prevalence (aPR = 1.38, 95% CI: 1.01–1.88). In both specifications, the inverse association between older age and self-medication persisted, indicating that joint pain and injury capture distinct experiential dimensions rather than reflecting mutual confounding (Table S5).

Third, adjusted predicted probabilities from the Poisson regression model were examined to aid interpretation. Predicted self-medication prevalence was higher among participants reporting fractures than among those with sprain or strain injuries (53.2% vs. 38.7%). A similar pattern was observed for joint pain, with higher predicted prevalence among those reporting joint pain compared with those without joint pain (52.8% vs. 40.4%) (Figure S1).

Finally, as an additional robustness check, the main Poisson model was re-estimated using cluster-robust standard errors at the village level (five clusters). Estimates were directionally consistent with the primary model: fracture remained significantly associated with higher self-medication prevalence, while joint pain showed a similar positive association of borderline statistical significance. Older age groups continued to show lower self-medication prevalence. Given the small number of clusters, these results are interpreted as a sensitivity analysis rather than the primary inferential model. Taken together, these sensitivity analyses demonstrate that the observed associations are robust to alternative modelling strategies, exclusion of correlated exposures, and clustering assumptions, strengthening confidence in the main findings.

## Discussion

### Summary of key findings

This study examined self-medication practices among older adults (≥ 55 years; *n* = 520) living in rural Tamil Nadu and identified several important patterns. Overall, 41.7% of participants reported self-medication in the past three months, indicating that unsupervised medicine use is a common component of everyday health management in later life. In adjusted analyses, chronic illness burden and multimorbidity were not significantly associated with self-medication, despite their high prevalence in the study population. In contrast, symptom- and function-related conditions—particularly recent injury (including fractures) and current joint pain—showed the strongest associations with self-medication. Inverse associations were observed among adults aged ≥ 60 years and among those currently married; these patterns may be consistent with differences in household support or health decision-making, although such mechanisms were not directly measured. A marginal positive association with primary education was also observed, suggesting that limited schooling may be sufficient to enable basic medication autonomy in some contexts. Importantly, self-medication did not differ significantly by gender, caste, religion, or household income, suggesting that in this rural setting self-medication may be shaped more by shared access constraints and symptom experiences than by sociodemographic stratification. Prevalence estimates are known to vary across studies by population, recall period, and setting, underscoring the importance of context-specific interpretation.

### Pain and injury versus chronic disease

Building on these findings, this study highlights a critical distinction between chronic disease status and pain- or injury-related conditions in shaping self-medication behaviour. A key contribution of this study lies in distinguishing between chronic disease status and pain- or injury-related conditions in shaping self-medication behaviour among older adults. Contrary to common assumptions, chronic morbidity did not independently predict self-medication in multivariable models. In contrast, pain-related conditions—particularly recent injury, fractures, and joint pain—were strongly associated with self-medication. Informal care-seeking among older adults may be driven less by diagnosed disease status and more by unmanaged pain and physical discomfort [54].

This finding aligns with nationally representative evidence showing that pain is highly prevalent among older adults in India but remains inadequately addressed within routine primary care. Data from the Longitudinal Ageing Study in India indicate that more than one-third of adults aged 45 years and older are often troubled by pain, with higher prevalence among women, rural residents, and socioeconomically disadvantaged groups [12]. Although injuries account for a smaller proportion of hospitalisations nationally, they frequently result in prolonged pain, reduced mobility, and delayed recovery—especially in rural settings where rehabilitative services and follow-up care are limited [13]. Together, self-medication in later life may be driven less by long-standing biomedical diagnoses and more by immediate, embodied experiences of discomfort and functional limitation. Disease-centred frameworks of healthcare utilisation may therefore underestimate the role of pain and injury as triggers for informal care-seeking. Recognising pain, physical discomfort, and functional decline as legitimate drivers of healthcare behaviour is essential for understanding self-medication practices among ageing populations.

### Intended versus realised care-seeking behaviour

An important insight from this study is the divergence between intended and realised care-seeking behaviour. While most participants reported that they would prefer to seek doctor-prescribed care as a first response to illness, a substantially higher proportion reported having self-medicated in the preceding three months. This gap suggests that care-seeking intentions are frequently overridden by situational constraints. High levels of reported financial barriers, transport difficulties, distance to facilities, and lack of accompaniment indicate that self-medication often functions as a supplementary strategy when formal care is delayed, inaccessible, or perceived as ineffective. Rather than reflecting a rejection of the health system, self-medication appears to represent episodic adaptation to structural constraints. Similar oscillation between formal and informal care has been documented in other low-resource settings, where care pathways are shaped by accessibility, symptom severity, and prior experiences with health services [55].

### Theoretical interpretation: Andersen’s model and structural vulnerability

Interpreted through Andersen’s Behavioral Model of Health Services Use, the findings highlight a disconnect between medically defined “need” (e.g., diagnosed chronic illness) and perceived urgency (e.g., pain, injury, functional impairment). In this study, perceived need—rooted in embodied discomfort—appears to exert a stronger influence on self-medication behaviour than diagnostic labels alone. This underscores the limitations of disease-centred public health frameworks and supports calls to incorporate experiential dimensions of ageing into definitions of healthcare need [56, 57]. The Structural Vulnerability Framework further clarifies why self-medication persists among rural older adults. Mobility limitations, financial hardship, transport barriers, and the absence of caregiver accompaniment operate as structural filters shaping everyday health decisions [58]. Within these constraints, self-medication emerges as a rational, albeit potentially risky, response rather than an expression of autonomy or convenience.

### Suggested pathways of self-medication among rural older adults

**Fig. 4:**
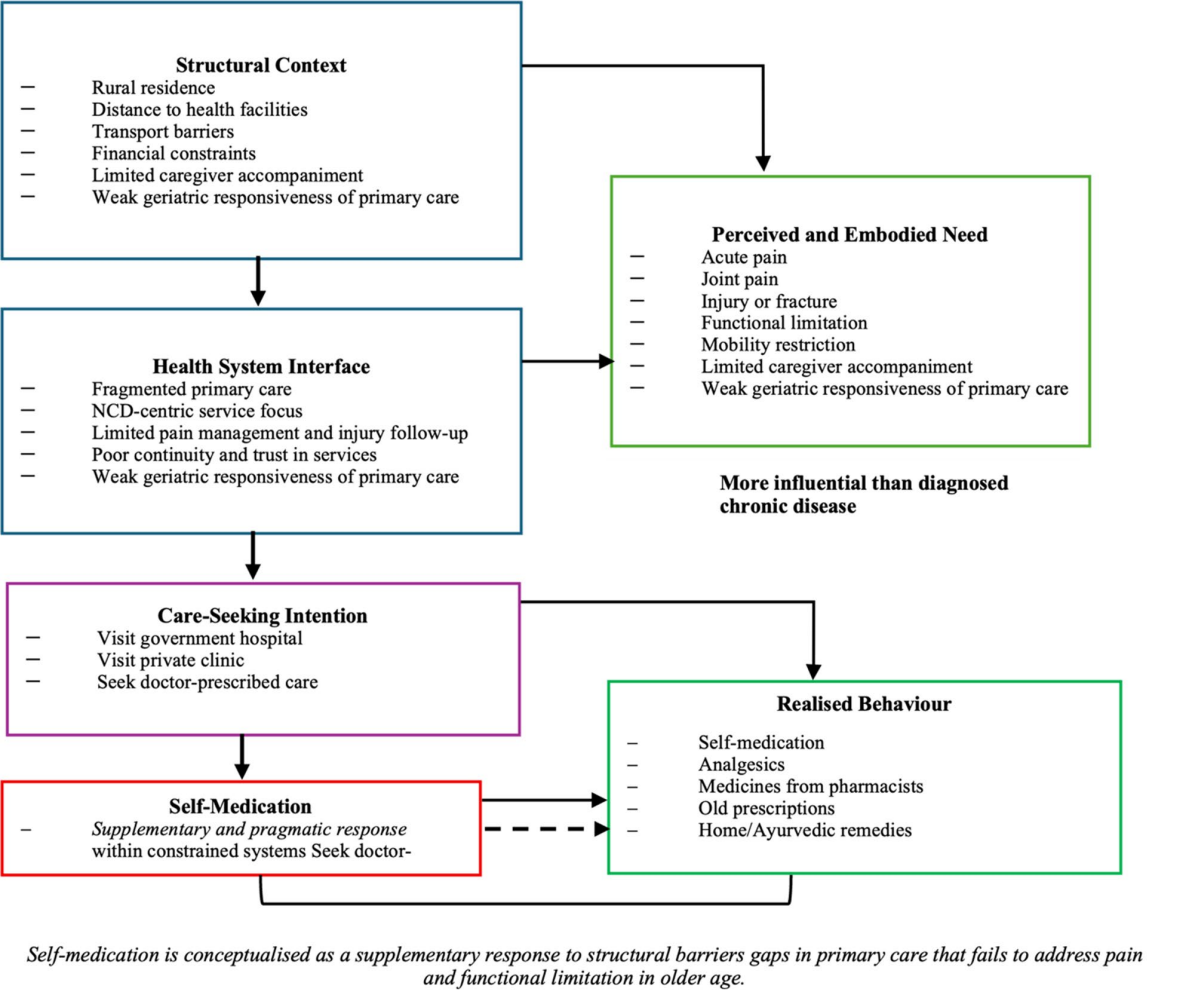
Pathways linking structural constraints, pain-related need, and self-medication among older adults in rural India. This conceptual pathway illustrates how structural barriers and gaps in geriatric-responsive primary care interact with pain and injury-related needs to shape self-medication among older adults. Self-medication is conceptualised as an adaptive, supplementary response that emerges when formal care does not adequately address embodied discomfort and functional limitation

This pathway is illustrated in Fig. 4, which situates self-medication within a sequence of structural constraints, system interactions, and embodied need. This pathway is not intended as a causal model but as an interpretive framework. Findings from this study suggest that self-medication among rural older adults emerges through a layered pathway shaped by structural, health system, and experiential factors rather than individual preference alone. At the upstream level, structural constraints—including rural residence, distance to health facilities, transport and financial barriers, limited caregiver accompaniment, and variable geriatric responsiveness of primary care—define what forms of care are feasible in everyday life. These conditions shape care options before individual decisions are made. At the health system interface, older adults encounter fragmented primary care characterised by an emphasis on NCD surveillance, limited attention to pain management and injury follow-up, and weak continuity of care. Although formal services are available, they often do not adequately respond to lived and functional health needs. Within this context, perceived and embodied needs—particularly acute pain, joint pain, injury, fracture, and mobility limitation—emerge as the primary triggers for action and are more influential than diagnosed chronic disease status. While stated care-seeking intentions commonly involve visiting government or private facilities and seeking doctor-prescribed care, these intentions are frequently disrupted by persistent symptoms and access constraints. Over time, this leads to realised behaviour in the form of self-medication, including the use of analgesics, medicines obtained from pharmacists, old prescriptions, and home or ayurvedic remedies. Importantly, self-medication in this pathway functions as a supplementary and pragmatic strategy within constrained systems rather than as disengagement from formal care. This interpretation aligns with Andersen’s emphasis on perceived need, the structural vulnerability framework, and a health equity perspective that situates self-medication as an adaptive response to gaps in system responsiveness.

### Health equity framing

From a health equity perspective, the absence of marked differences in self-medication by gender, caste, religion, or household income is noteworthy. Rather than indicating the absence of inequity, this pattern suggests that older adults across social groups in rural settings may be similarly exposed to common health system constraints. In such contexts, inequities appear to operate less through individual social position and more through features of service design, availability, and responsiveness. Primary care systems in India have made important progress in expanding coverage for major non-communicable diseases (NCDs). However, their operational emphasis often remains focused on diagnosis and surveillance, with comparatively less attention to pain management, functional limitations, and follow-up care in later life. Older adults whose health needs are episodic, pain-driven, or mobility-related may therefore find fewer entry points into routine care pathways. The persistence of self-medication even among individuals with existing prescriptions in this study may reflect gaps in continuity of care, medication counselling, and follow-up support rather than disengagement from formal services. Conditions such as thyroid disorders or respiratory symptoms may remain incompletely managed not only because they are less visible within programmatic priorities, but also because repeated care-seeking may be perceived as burdensome or of limited benefit, particularly when services are distant or difficult to access [59, 60]. In this sense, self-medication appears to function as a compensatory strategy within constrained care environments. Viewing self-medication through a health equity lens therefore shifts the focus away from individual behaviour alone and toward opportunities for system strengthening. It highlights the importance of primary care models that are more responsive. Such an approach aligns with ongoing health system reforms and offers a pathway to support safer care-seeking practices among older adults by addressing underlying access, continuity, and responsiveness gaps.

### Policy and programmatic implications

This study points to important opportunities for India’s rural primary healthcare system to more fully respond to the evolving health needs of older adults. Particularly in relation to injury-related pain, musculoskeletal conditions, and continuity of care. National initiatives such as Ayushman Bharat and Health and Wellness Centres (HWCs) have substantially expanded access to essential services and strengthened the primary care platform. However, their operational emphasis has largely focused on formal diagnoses and priority non-communicable diseases (NCDs) [61]. Emerging evidence suggests that the extent to which HWCs address the everyday, function-limiting health concerns of older adults remains uneven and insufficiently documented [62]. As a result, conditions such as joint pain, injury-related discomfort, and functional decline—while highly prevalent and impactful for older adults—may receive less systematic attention within routine primary care workflows [63]. The elevated prevalence of self-medication among participants reporting fractures and joint pain in this study highlights the importance of strengthening pain management and injury follow-up within existing service delivery structures. Despite their influence on mobility, independence, and quality of life, these conditions are not always integrated into standard care packages, reflecting long-standing clinical and programmatic emphases to prioritise disease control over symptom relief and functional wellbeing [10, 11, 20, 22].

Self-medication in this context appears to arise from a combination of factors rather than unmet health needs alone. It is shaped in part by locally embedded healing practices and norms of self-care that coexist with formal healthcare systems in rural settings [64]. At the same time, structural constraints—including financial pressures, mobility limitations, transport challenges, and variable trust in public-sector services. These factors continue to influence care-seeking decisions among older adults. Even as insurance coverage expands, out-of-pocket costs, logistical barriers, and the absence of caregiving support may limit timely and sustained engagement with formal care [55, 59, 60, 65]. Provider communication and perceptions of service quality also play an important role in shaping older adults’ willingness to seek follow-up care [56, 57, 66, 67]. Advancing equitable and age-responsive primary health care for rural populations therefore requires a gradual shift from predominantly disease-centred metrics toward more needs-responsive and function-sensitive care models. Based on the findings of this study, several programmatic directions warrant consideration:

- Integrating community-based screening and follow-up for musculoskeletal injuries and joint pain within routine HWC outreach and geriatric assessments [41, 58];
- Embedding medication safety counselling, including guidance on polypharmacy and unsupervised drug use, into HWC and Aayush Arogya Mandir services [41];
- Expanding mobile health units and home-based outreach to address mobility limitations and geographic barriers in remote and underserved rural areas [66, 67];
- Strengthening geriatric capacity among frontline health workers, with focused training in pain recognition, frailty screening, and care for socially vulnerable older adults [68, 69];

Such efforts are most likely to be effective when grounded in participatory, context-sensitive planning that incorporates the lived experiences of older adults and frontline providers. Broadening what is defined, recognised, and resourced as a “health need” can help primary care systems better support safe, timely, and appropriate care-seeking in later life [70]. These directions align closely with India’s Universal Health Coverage (UHC) roadmap and contribute to progress toward key Sustainable Development Goals, particularly SDG 3.8 (access to quality essential health services) and SDG 10 (reducing inequality) [71, 72]. Strengthening pain-sensitive and age-inclusive care models thus represents both a national priority and a meaningful step toward advancing health equity for older adults.

## Strengths and limitations

This study contributes community-based evidence on self-medication among older adults living in rural India, a population that remains under-represented in existing research. Inclusion of adults aged 55–59 years captures a transitional stage of ageing that is frequently overlooked in national analyses, despite emerging multimorbidity, pain, and changes in help-seeking behaviour. By foregrounding pain, injury, and functional discomfort rather than chronic disease status alone, the study offers a more nuanced understanding of experiential drivers of informal and unsupervised medication use in later life. Methodological strengths include structured interviewer training, standardised digital data collection with built-in quality checks, and multiple sensitivity analyses that support the robustness of observed associations.

Several limitations should be acknowledged. The cross-sectional design precludes causal inference. Self-reported measures may be subject to recall bias, and medication use was not clinically verified through pill packets or prescription review; formal medication adherence and appropriateness were also not assessed. Older adults unable to provide informed consent due to apparent severe cognitive impairment or functional limitations were excluded, and proxy interviews were not conducted, which may have led to under-representation of the most frail. Although missing data were minimal and non-systematic, analyses were based on complete cases. Data collection occurred between May and August, and seasonal factors such as agricultural work patterns, heat, and intermittent weather-related mobility constraints may have influenced household availability and participation. Finally, the study was conducted in a limited geographic area, which may constrain generalisability to other rural settings. Despite these limitations, the findings provide policy-relevant insights into how structural constraints and symptom-driven health needs shape self-medication practices among older adults in rural contexts.

## Conclusion

Self-medication is highly prevalent among older adults in rural Tamil Nadu and is more strongly associated with pain and injury than with diagnosed chronic disease. The findings challenge portrayals of self-medication as reckless behaviour and instead position it as an adaptive response to gaps in pain management, injury follow-up, and continuity of care. Addressing self-medication in later life therefore requires moving beyond behaviour-focused narratives toward strengthening geriatric-responsive, equitable, and pain-sensitive primary care. Without such reforms, informal medication practices will continue to function as necessary—but potentially unsafe—substitutes for accessible and responsive care in ageing rural populations.

## Supplementary Information

Below is the link to the electronic supplementary material.

Supplementary Material 1

## Data Availability

The dataset generated and analyzed during the current study is available on the Open Science Framework (OSF) repository (https://osf.io/bkyas). The dataset has been de-identified to protect participant confidentiality and is shared in accordance with institutional ethical approvals. Additional supporting materials (e.g., instruments, codebooks) may be made available upon reasonable request to the corresponding author.

## Abbreviations

aOR: Adjusted odds ratio
aPR: Adjusted prevalence ratio
AIIMS: All India Institute of Medical Sciences
AWW: Anganwadi Worker
AB–HWC: Ayushman Bharat – Health and Wellness Centre
HWC: Health and Wellness Centre
BC: Backward Classes
χ²: Chi-square test
CI: Confidence interval
CS: Cross-sectional study
NCD: Current non-communicable disease
FBT: Forward–backward translation
GLM: Generalized linear model
GoI: Government of India
HWC: Health and Wellness Centre
IIPS: Indian Institute of Population Sciences
t-test: Independent-samples t-test
LASI: Longitudinal Ageing Study in India
LMICs: Low- and middle-income countries
MPH: Master of Public Health
MBC: Most Backward Class
Multimorbidity: Multicentre or multi-morbidity
OBC: Other Backward Class
OPD: Outpatient department
χ²: Pearson’s chi-square test
PR: Prevalence ratio
PHC: Primary health centre
PM-JAY: Pradhan Mantri Jan Arogya Yojana
PIB: Public Information Bureau
RPU: Rural–peri-urban interface
SC: Scheduled Caste
ST: Scheduled Tribe
SD: Standard deviation
UHC: Universal health coverage
UBA: Unnat Bharat Abhiyan
VIF: Variance inflation factor
WHO: World Health Organization
