# Supplementary material for: Self-medication among older adults in rural India: structural gaps in primary care and pain management
Source: BMC Health Serv Res. 2026 Feb 5;26:333. doi: 10.1186/s12913-026-14114-z (PMC12964660; doi:10.1186/s12913-026-14114-z)
Supplement: Supplementary file 1 — Supplementary Material 1 [file 12913_2026_14114_MOESM1_ESM.docx]

**Supplementary Tables and Figures**

**Table S1** summarises self-reported structural barriers to healthcare access among older adults. Financial constraints, distance to health facilities, and transport-related difficulties were the most commonly reported barriers, while a smaller proportion reported social barriers such as lack of accompaniment. These barriers are not mutually exclusive and may co-occur, compounding challenges in timely care-seeking.

### ****Table S1. Reported structural barriers to accessing healthcare among older adults (N = 519)****

| Barrier to healthcare access | Category | n | % |
| --- | --- | --- | --- |
| **Distance to health facility** | No | 347 | 66.9 |
|  | Yes | 172 | 33.1 |
| **Financial constraints** | No | 299 | 57.6 |
|  | Yes | 220 | 42.4 |
| **Transport-related difficulties** | No | 343 | 66.1 |
|  | Yes | 176 | 33.9 |
| **No one available to accompany** | No | 429 | 82.7 |
|  | Yes | 90 | 17.3 |
| **No perceived access-related concerns** | No | 369 | 71.1 |
|  | Yes | 150 | 28.9 |
| **Other concerns†** | No | 488 | 94.0 |
|  | Yes | 31 | 6.0 |

### **Footnotes.**

- *Percentages are calculated using valid responses; one participant had missing data for each barrier item (valid* N *= 519 per row).*
- *Participants could report multiple barriers; categories are therefore not mutually exclusive.*
- *Barriers were self-reported and reflect perceived challenges in accessing healthcare services.*
- *†“Other concerns” included responses not captured by predefined categories, such as long waiting times and perceived quality of care.*

**Table S2** summarises model diagnostics and robustness checks for the multivariable analyses. Missing data were minimal, multicollinearity was negligible, and model fit was adequate. Sensitivity analyses comparing Poisson and logistic regression models yielded directionally consistent estimates, supporting the robustness of the findings.

**Table S2. Model diagnostics and robustness checks for multivariable analyses (N = 520)**

| Diagnostic domain | Indicator | Result | Interpretation |
| --- | --- | --- | --- |
| Missing data | Participants with complete data for outcome and all covariates | 505 / 520 (97.1%) | Minimal missingness; complete-case analysis justified |
|  | Participants excluded due to missing data | 15 / 520  (2.9%) | Missingness primarily due to chronic illness status (n = 14) or injury type  (n = 1) |
| Collinearity | Mean variance inflation factor (VIF) | 1.36 | No evidence of multicollinearity |
|  | Range of VIFs | 1.05 – 1.76 | All values well below conventional thresholds (VIF < 5) |
|  | Joint pain VIF | 1.05 | Minimal correlation with injury type |
|  | Injury type VIF | 1.11 | Minimal correlation with joint pain |
| Model fit | Pearson goodness-of-fit χ² (Poisson model) | χ² = 295.9;  df = 484;  p = 1.00 | Adequate model fit; no evidence of overdispersion |
| Outcome events | Number of self-medication events | 209 | Sufficient outcome prevalence for regression modelling |
| Model complexity | Number of non-intercept predictors | 34 | Includes categorical covariates |
|  | Events per variable (EPV) | 6.1 | Acceptable for exploratory multivariable models with robust standard errors |
| Sensitivity analysis | Logistic regression (aOR) vs Poisson regression (aPR) | Directionally consistent | Findings robust to model specification |

### **Footnotes.**

- *Diagnostics are based on the complete-case analytic sample used in the primary Poisson regression model with robust standard errors.*
- *Variance inflation factors (VIFs) were calculated using an ordinary least squares regression including all covariates from the main multivariable model.*
- *Although the events-per-variable (EPV) value was slightly below conservative thresholds (≥10), estimates were considered acceptable given the use of robust variance estimation and consistency of results across sensitivity analyses.*

**Table S3** presents a bivariate cross-tabulation of joint pain and injury type to assess potential collinearity. While a statistically significant association was observed, the small effect size (Cramér’s V = 0.096) indicates weak dependence, supporting the inclusion of both variables in multivariable analyses.

### ****Table S3. Collinearity check between joint pain and injury type (sprain/strain vs fracture)****

| Joint pain status | Sprain/strain | Fracture | Total | Pearson χ² | Fisher’s exact test | Effect size: |
| --- | --- | --- | --- | --- | --- | --- |
| No joint pain | 396 | 86 | 482 | (df = 1) = 4.78,  p = 0.029 | p = 0.046 | Cramér’s V (2 × 2) = 0.096 (small) |
| Joint pain | 25 | 12 | 37 |  |  |  |
| **Total** | **421** | **98** | **519** |  |  |  |

### **Footnotes.**

- *Values are frequencies (*n*).*
- *Although the association between joint pain and injury type was statistically significant, the effect size was small, indicating limited overlap between these variables.*
- *This supports their simultaneous inclusion as independent predictors in multivariable regression models without concerns of problematic collinearity.*

**Table S4** presents unadjusted and adjusted associations with self-medication in the past three months. Fracture in the past year and younger age were independently associated with higher self-medication prevalence in adjusted analyses, while chronic illness burden per se was not, underscoring the importance of acute injury and life-stage factors in shaping unsupervised medicine use among older adults.

**Table S4. Associations with self-medication in the past 3 months: unadjusted and adjusted analyses**

| **Predictor** | **Category (vs reference)** | **Bivariable OR (95% CI)** | ***p*** | ***N*** | **Adjusted aOR (95% CI)** | ***p*** | **Adjusted aPR (95% CI)** | ***p*** |
| --- | --- | --- | --- | --- | --- | --- | --- | --- |
| Chronic illness | Single (vs none) | 0.84  (0.54–1.29) | 0.424 | 506 | 0.89  (0.55–1.42) | 0.615 | 0.93  (0.72–1.20) | 0.575 |
|  | Co-/multi-morbidity (vs none) | 0.71  (0.45–1.11) | 0.135 | 506 | 0.74  (0.45–1.20) | 0.217 | 0.85  (0.65–1.10) | 0.216 |
| Joint pain | Yes (vs no) | 2.17  (1.10–4.28) | 0.026 | 520 | 1.82  (0.89–3.73) | 0.102 | 1.31  (0.96–1.78) | 0.087 |
| Injury in past 12 months | Fracture (vs sprain/strain) | 1.75  (1.13–2.73) | 0.013 | 519 | 1.87  (1.14–3.07) | 0.013 | 1.38  (1.09–1.74) | 0.007 |
| Gender | Female (vs male) | 0.97  (0.66–1.42) | 0.886 | 520 | 0.91  (0.54–1.52) | 0.706 | 0.96  (0.73–1.26) | 0.767 |
| Age group | 60–74 (vs 55–59) | 0.63  (0.41–0.98) | 0.041 | 520 | 0.56  (0.35–0.92) | 0.022 | 0.74  (0.58–0.96) | 0.021 |
|  | ≥75 (vs 55–59) | 0.67  (0.37–1.20) | 0.179 | 520 | 0.50  (0.25–0.99) | 0.046 | 0.69  (0.48–1.00) | 0.052 |
| Marital/union status | Currently in union (vs not) | 0.69  (0.47–1.00) | 0.047 | 520 | 0.69  (0.44–1.08) | 0.101 | 0.83  (0.65–1.05) | 0.114 |
| Living arrangement | With family/others (vs alone) | 0.82  (0.46–1.46) | 0.496 | 520 | 0.88  (0.45–1.70) | 0.696 | 0.94  (0.67–1.31) | 0.701 |
| Religion | Muslim (vs Hindu) | 0.81  (0.31–2.11) | 0.673 | 520 | 1.02  (0.33–3.12) | 0.976 | 0.98  (0.55–1.74) | 0.941 |
|  | Christian (vs Hindu) | 1.07  (0.62–1.84) | 0.813 | 520 | 1.22  (0.66–2.26) | 0.525 | 1.11  (0.81–1.54) | 0.507 |
| Caste | OBC/MBC (vs SC/ST) | 1.26  (0.87–1.83) | 0.225 | 520 | 1.34  (0.86–2.07) | 0.192 | 1.17  (0.91–1.51) | 0.225 |
|  | Other/None (vs SC/ST) | 0.80  (0.32–1.96) | 0.622 | 520 | 0.60  (0.21–1.75) | 0.352 | 0.75  (0.38–1.49) | 0.410 |
| Education | 1–5th (vs none) | 1.49  (0.97–2.31) | 0.071 | 520 | 1.55  (0.95–2.53) | 0.076 | 1.28  (0.98–1.66) | 0.070 |
|  | ≥6th (vs none) | 1.01  (0.67–1.53) | 0.959 | 520 | 1.02  (0.62–1.66) | 0.946 | 1.02  (0.77–1.36) | 0.896 |
| Worked in past 12 months | Yes (vs no) | 0.80  (0.56–1.14) | 0.212 | 520 | 0.82  (0.55–1.22) | 0.333 | 0.90  (0.72–1.12) | 0.346 |
| Monthly income | ₹5,001–10,000 (vs ≤₹5,000) | 0.94  (0.63–1.41) | 0.773 | 52 |  |  |  |  |

### **Footnotes.**

- ***Outcome:*** *Any self-medication in the past 3 months (binary)*
- ***Bivariable models:*** *Robust standard errors*
- ***Adjusted models:*** *Complete-case analysis (*N *= 505)*
- ***Reference categories:*** *Shown in parentheses*
- *Adjusted prevalence ratios (aPRs) were estimated using Poisson regression with robust standard errors.*
- *Adjusted odds ratios (aORs) were estimated using fully adjusted logistic regression on the same complete-case sample (*N *= 505).*
- *Bivariable models were estimated separately for each predictor and may have slightly different* N *due to item-level missingness.*
- *Reference categories are indicated in parentheses.*

**Table S5** presents sensitivity analyses evaluating the robustness of associations between injury type, joint pain, and self-medication. Effect estimates for fracture remained stable when joint pain was excluded, and joint pain became statistically significant when injury type was excluded, indicating partial correlation but independent contributions of acute injury and pain-related pathways to self-medication behaviour.

**Table S5. Sensitivity analyses assessing robustness of Poisson models to correlated exposures (N = 505)**

| Model specification | Key exposure | Adjusted PR (95% CI) | *p*-value |
| --- | --- | --- | --- |
| Main model | Fracture (vs sprain/strain) | 1.38 (1.09–1.74) | 0.007 |
|  | Joint pain (yes vs no) | 1.31 (0.96–1.78) | 0.087 |
| Model excluding joint pain | Fracture (vs sprain/strain) | 1.41 (1.12–1.78) | 0.004 |
| Model excluding injury type | Joint pain (yes vs no) | 1.38 (1.01–1.88) | 0.041 |

### **Footnotes.**

- *Estimates are adjusted prevalence ratios (PRs) derived from Poisson regression models with robust standard errors.*
- *All models were adjusted for chronic illness status, sex, age group, marital/union status, living arrangement, religion, caste, education, work status, monthly income, tobacco use, and alcohol use.*
- *Analyses were restricted to the complete-case analytic sample (*N *= 505).*

### ****Figure S1. Adjusted predicted probability of self-medication.**** Adjusted predicted probabilities of any self-medication in the past three months estimated from Poisson regression models with robust standard errors (N = 505). **Panel A** shows predicted probabilities by injury type in the past 12 months (sprain/strain vs. fracture), and **Panel B** shows predicted probabilities by joint pain status (no vs. yes). Points represent adjusted predicted probabilities and vertical bars indicate 95% confidence intervals. Models were adjusted for chronic illness status, sex, age group, marital/union status, living arrangement, religion, caste, education, work status in the past 12 months, household income, tobacco use, and alcohol use.

***
